# The Inhibition of miR-873 Provides Therapeutic Benefit in a Lipopolysaccharide-Induced Neuroinflammatory Model of Parkinson's Disease

**DOI:** 10.1155/2020/8735249

**Published:** 2020-07-15

**Authors:** Jinhua Wu, Xuming Yu, Ke Xue, Juan Wu, Rongyan Wang, Xianfei Xie, Ke Li, Zheqiong Yang, Jiang Yue

**Affiliations:** ^1^Department of Pharmacology, Wuhan University School of Basic Medical Sciences, Wuhan 430071, China; ^2^Reproductive Medicine Center, Taihe Hospital, Hubei University of Medicine, Shiyan 442000, China; ^3^Demonstration Center for Experimental Basic Medicine Education, School of Basic Medical Sciences, Wuhan University, Wuhan 430071, China; ^4^Hubei Province Key Laboratory of Allergy and Immunology, Wuhan 430060, China

## Abstract

*Background and Purpose.* Alterations in cholesterol homeostasis have been reported in cell and animal models of Parkinson's disease (PD), although there are inconsistent data about the association between serum cholesterol levels and risk of PD. Here, we investigated the effects of miR-873 on lysosomal cholesterol homeostasis and progressive dopaminergic neuron damage in a lipopolysaccharide-(LPS) induced model of PD. *Experimental Approach*. To evaluate the therapeutic benefit of the miR-873 sponge, rats were injected with a LV-miR-873 sponge or the control vector 3 days before the right-unilateral injection of LPS into the substantia nigra (SN) pars compacta, or 8 and 16 days after LPS injection. Normal SH-SY5Y cells or SH-SY5Y cells overexpressing *α*-synuclein were used to evaluate the distribution of *α*-synuclein and cholesterol in lysosomes and to assess the autophagic flux after miR-873 transfection or ABCA1 silencing. The inhibition of miR-873 significantly ameliorated the LPS-induced accumulation of *α*-synuclein and loss of dopaminergic neurons in the SN at the early stage. miR-873 mediated the inhibition of ABCA1 by LPS. miR-873 transfection or ABCA1 silencing increased the lysosomal cholesterol and *α*-synuclein levels, and decreased the autophagic flux. The knockdown of ABCA1 or A20, which are the downstream target genes of miR-873, exacerbated the damage to LPS-induced dopaminergic neurons. *Conclusion and Implications*. The results suggest that the inhibition of miR-873 may play a dual protective role by improving intracellular cholesterol homeostasis and neuroinflammation in PD. The therapeutic effects of the miR-873 sponge in PD may be due to the upregulation of ABCA1 and A20.

## 1. Introduction

Parkinson's disease (PD) is the second most common neurodegenerative disorder, and its pathological changes are characterized by the accumulation of *α*-synuclein within Lewy bodies and the loss of dopamine (DA) neurons in the substantia nigra (SN). More recent data suggest that the main mechanism of *α*-synuclein clearance is lysosome-dependent autophagy, although multiple mechanisms are involved in the degradation of *α*-synuclein including the ubiquitin proteasome pathway [1].

Cholesterol, which is particularly enriched in the brain, participates in key structural and physiological functions, including synaptic vesicle formation and membrane trafficking [2]. Alterations in cholesterol homeostasis have been reported in cell or animal models of PD, although there are inconsistent data about the association between serum cholesterol levels and risk of PD [3]. A previous study showed that the accumulation of cholesterol in a lysosomal-like pattern was observed in pre-apoptotic cells treated with the neurotoxin 1-methyl-4-phenylpyridinium (MPP+), and high cholesterol also induced the accumulation of *α*-synuclein [4].

Human ATP-binding cassette transporter A1 (ABCA1) plays a critical role in mediating both intercellular and cellular cholesterol efflux. Previous data revealed a significant decrease in the length of neurites and the number of neurite segments in the hippocampal CA1 region of ABCA1 knockout mice compared to those in the hippocampal CA1 region of wild-type mice, suggesting the importance of ABCA1 disruption for neurite degeneration in the brain [5]. ABCA1 knockout mice had a reduced number of synapses in the cortex and a significantly reduced number of vesicles in the presynaptic and posterior neurons [6]. Niemann-Pick disease type C (NPC), which is caused by mutations in the NPC1 or NPC2 genes, is a progressive neurodegenerative disease that is characterized by the accumulation of unesterified cholesterol in late endosomes and lysosomes [7]. The selective upregulation of ABCA1 in NPC1^−/−^ cells can correct the efflux of free cholesterol from lysosomes and increase cholesterol levels that are available for esterification, indicating a key role of ABCA1 in the mobilization of intracellular cholesterol [8]. Although the role of ABCA1 in neurodegenerative disorders has not been elucidated, ABCA1 has been shown to affect the susceptibility of the central nervous system to inflammation and neuronal death. A previous study showed that ABCA1 deficiency in 90% of the brain cells resulted in significant microglia activation [9], which is often associated with increased Toll-like receptor signaling [10].

A wide variety of data indicate that neuroinflammation plays a critical role in PD [11]. Recent studies indicated that miRNAs can elevate a pro-inflammatory response via the downregulation of anti-inflammatory proteins and can be involved in the pathologic processes of spinal cord injury, multiple sclerosis, ischemic stroke, and Alzheimer's disease [12]. A previous study showed that miR-873 silencing improved the neuroinflammation and demyelination in experimental autoimmune encephalomyelitis mice [13]. Online databases predict that ABCA1 is a target gene of miR-873. The injection of lipopolysaccharide (LPS) into the SN region results in a progressive, specific, and irreversible loss of DA neurons and accumulation of *α*-synuclein [14]. The present study is aimed at investigating the effects of miR-873 on cholesterol homeostasis via ABCA1 and progressive DA neuron damage using a LPS-induced neuroinflammatory model of Parkinson's disease.

## 2. Methods

### 2.1. Animals

Male adult Wistar rats (250-300 g) were supplied by the Experimental Animal Center (Hubei, China). The animals were kept in a room (22-25°C) with a 12 h artificial light/dark cycle and had free access to food and water. All the animal care and experimental procedures were approved by the Animal Care Committee of Wuhan University and complied with the recommendations of the International Association for the Study of Pain. All the studies involving animals are reported in accordance with the ARRIVE guidelines for reporting experiments involving animals [15, 16].

### 2.2. Lentiviral Vector (LV) and Plasmid Construction

The miR-873 sponge and shRNAs targeting ABCA1 and A20 were purchased (Longqian Biotech, Shanghai, China) and cloned into the lentiviral pHAGE-CMV-MCS-IZsGreen vector. The binding sequence of miR-873 was as follows: AGGAGACTGATGTTCCTGC. The sequence of the shRNA targeting human ABCA1 mRNA was as follows: TAGTCCTCTTTCCCGCATTAT. The sequence of the shRNA targeting rat ABCA1 mRNA was as follows: GACCACCCTAGAAGAAATATT. The sequence of the shRNA targeting rat A20 mRNA was as follows: CTTGTTCAGCACGAATACAAG. The expression vectors carrying the miR-873 sponge, sh-ABCA1, or sh-A20 or the control vector were transfected with packing and envelope plasmids (psPAX2 and pMD2.G; Cambridge, MA, USA) into 293T cells using Lipofectamine™ 2000 (Invitrogen, Carlsbad, CA, USA). The virus-containing medium was harvested 60 h later and was concentrated by a two-step ultracentrifugation procedure after the filtration. The viral vectors were mixed, and the final titer of the viral vectors was 2 × 10^9^ TU/ml. The viral stocks were stored at -80°C until use. The animals were injected with 1 *μ*l of viral vectors.

Fragments encoding primary miR-873 were cloned into the pcDNA-3.1(-) vector (Invitrogen, Carlsbad, CA, USA) following digestion with Xhol/HindIII (Thermo Scientific, Waltham, MA, USA). Fragments encoding *α*-synuclein were cloned into a pHAGE-CMV-MCS-IZsGreen vector containing the enhanced green fluorescent protein (eGFP) gene (Stargene Sci-Tech Development, Wuhan, China) after the digestion with NotI/XbaI (Thermo Scientific). To construct the luciferase reporter plasmids, the ABCA1 3′-untranslated region (3′-UTR) was cloned into the pMiR-report vector following digestion with Sacl/HindIII (Thermo Scientific). All the final constructs were verified by DNA sequence analysis.

### 2.3. Neurosurgery and Treatment

The procedure for the implantation of the guide cannula was performed as previously described [17]. The rats were anaesthetized by intraperitoneal injection with sodium pentobarbital (50 mg/kg, i.p.) and were secured in a stereotaxic frame (RWD Life Science, Shenzhen, China). A guide cannula (62003, RWD Life Science) was implanted 0.5 mm above the right substantia nigra pars compacta (SNpc) (bregma coordinates: anterior-posterior—5.3 mm; medial-lateral—2.0 mm; and dorsal-ventral—7.8 mm), and the insertion cannula for stereotaxic injection protruded 0.5 mm below the tip of the guide cannula. Acrylic dental cement was used to fix the guide cannula with three stainless steel screws, and then the incision was closed. The animals were administered benzylpenicillin (60 mg/kg, s.c.) after the operation and were kept warm until they awakened. During the postsurgical recovery, the body weights and clinical signs of the rats were closely monitored. After 6 days of recovery, the rats were randomly divided into the different groups. All the rats were sacrificed after behavioral testing. For mRNA detection, the brains were quickly removed after the rats were decapitated. For immunohistochemistry, the animals were deeply anesthetized and then perfused with saline through the aorta followed by cold 4% paraformaldehyde. Then, the brain tissues were fixed with 4% paraformaldehyde.

LPS (from *Escherichia coli*, serotype O55:B5, Sigma-Aldrich, St. Louis, MO, USA) dissolved in phosphate-buffered saline (PBS) was injected (10 *μ*g, 2 *μ*l) into the SNpc of the rats at a rate of 0.2 *μ*l/min by an automatic injector (CMA 402, CMA Microdialysis AB, Sweden) [18, 19]. The syringe was left in situ for 5 min. The control animals received sterile PBS after the surgical procedures. To evaluate the therapeutic benefit of the miR-873 sponge in the LPS-induced model of PD, the rats received unilateral injections of the LV-miR-873 sponge or the control vector 3 days before LPS treatment or 8 and 16 days after LPS treatment. The expression of GFP was used to identify the injection site and to analyze LV transduction.

To investigate whether ABCA1 is involved in the development of PD, the rats received were unilaterally injected with the LV-sh-ABCA1 vector (1 *μ*l) or the LV-sh-control vector 3 days before LPS treatment. The LV vector was injected for 5 min at a rate of 0.2 *μ*l/min by an automatic injector. To evaluate the effects of A20 on the LPS-induced damage to DA neurons, the rats were unilaterally injected with LV-sh-A20 or the control vector 3 days before the stereotactic injection of LPS.

### 2.4. Rotational Behavior

Rotation induced by apomorphine is an indirect measure of the loss of striatal DA neurotransmission, and this assessment is widely used in animal studies of PD [20]. The rats were injected with the DA receptor agonist apomorphine (0.5 mg/kg, s.c.) dissolved in sterile saline 16 or 21 days after LPS injection to examine their rotational behaviors [21, 22]. The animals were placed in a circular test arena, and their rotational activities were measured 5 min after the injection. The number of turns was counted for 30 min.

### 2.5. Immunohistochemistry

For the SN region, four sections from each brain were selected from a series of 4 *μ*m sections, and the sections from the different groups were matched as closely as possible [23]. To label the DA neurons, the samples were incubated with a polyclonal rabbit anti-rat tyrosine hydroxylase (TH) antibody (1 : 200, Cat. GTX113016; GeneTex Inc., Irvine, CA, USA). The samples were incubated with biotinylated anti-rabbit IgG (1 : 100, Vector Laboratories, Burlington, Canada) and then visualized using the avidin-biotin complex technique (ABC kit, Vector Laboratories, Burlington, Canada) followed by the reaction with 3,3′-diaminobenzidine and hydrogen peroxide (DAB kit, Vector Laboratories, Burlington, Canada). The TH-positive cells in both the lesioned and intact hemispheres were automatically analyzed by ImageJ software (National Institutes of Health, Bethesda, MD, USA), and the data are expressed as a percentage of the value of the intact SN hemisphere.

### 2.6. Fluorescent Immunohistochemistry

The SN sections from the rats were co-incubated with a monoclonal mouse anti-rat *α*-synuclein antibody (1 : 400, Cat. ab1903; Abcam) and a polyclonal rabbit anti-rat TH antibody (1 : 200, Cat. GTX113016; GeneTex Inc.). The secondary antibodies were a DyLight 405-conjugated donkey anti-mouse IgG antibody (1 : 100, Wuhan Antgene Biotechnology) and a TRITC-conjugated goat anti-rabbit IgG antibody (1 : 100, Kerui Biotechnology). The images were captured by an Olympus BX51 fluorescence microscope (Olympus Corporation, Tokyo, Japan) equipped with an Olympus DP72 microscope digital camera. Identical illumination and camera settings were used to capture and save all the images.

### 2.7. Real-Time RT-PCR

Total RNA was isolated from the cells or SN regions using TRIzol Reagent (Invitrogen, Carlsbad, CA, USA) according to the manufacturer's protocol. For the first-strand synthesis, a cDNA Synthesis Kit (Toyobo, Osaka, Japan) was used to synthesize the cDNA. All the real-time PCR were performed with SYBR Green (Toyobo, Osaka, Japan) on a CFX Connect Real-Time PCR Detection System (Bio-Rad, Inc., Hercules, CA, USA). The primers and PCR conditions for the amplification of miR-873, pre-miR-873, ABCA1, A20, GCase, cathepsin D (CTSD), NPC1 and NPC2 are listed in Table S1. Human GAPDH was used to normalize the gene expression levels, including the levels of ABCA1, A20, GCase, CTSD, NPC1 and NPC2. U6 was used to normalize the relative expression of miR-873. The gene expression levels were calculated using the 2^−ΔΔCT^ method relative to the internal control.

### 2.8. Cell Culture and Treatment

Human glioma U251 and U87 cells and neuroblastoma SH-SY5Y cells were grown in Dulbecco's modified Eagle medium containing 10% fetal bovine serum. For the time course study, the expression levels of miR-873, pre-miR-873, and ABCA1 in U251 cells were determined after the incubation with LPS (100 ng/ml) for 24 h. To determine the involvement of the TLR4-MyD88 signaling pathway in the LPS-induced regulation of the miR-873 and ABCA1 levels, U251 cells were pretreated with or without selective antagonists of TLR4 (CLI-095, 1 *μ*M) or MyD88 (ST2825, 20 *μ*M) for 1 h before incubation with LPS for 24 h. U251 cells were transfected with miR-873 expression vector, miR-873 sponge, or empty vector for 6 h and then incubated with LPS for 24 h to investigate the involvement of miR-873 in the regulation of ABCA1 by LPS.

### 2.9. Immunoblotting

To detect the ABCA1 proteins, 25 *μ*g of total protein from U251 cells was separated by SDS-polyacrylamide gel electrophoresis (4% stacking and 10% resolving gels) and then transferred onto PVDF membranes overnight. These membranes were incubated with a polyclonal rabbit anti-human ABCA1 antibody (1 : 1000, NB400-105, Novus Biologicals, Colorado, USA) at 4°C overnight and then incubated with a biotinylated anti-rabbit IgG antibody (1 : 100, Vector Laboratories, Burlington, Canada). The protein levels of ABCA1 were normalized to those of *β*-actin (1 : 1000, Cat. sc-8432, Santa Cruz Biotechnology, Inc.) to control for the loading efficiency.

To detect the p62 and LC3 proteins, 25 *μ*g of total protein from SH-SY5Y cells was separated by SDS-polyacrylamide gel electrophoresis (4% stacking and 12% resolving gels) and then transferred onto PVDF membranes overnight. These membranes were incubated with a monoclonal rabbit anti-human LC3A/B antibody (1 : 1000, #12741S, Cell Signaling Technology, USA) or a polyclonal rabbit anti-human p62 antibody (1 : 1000, A7758, ABclonal, China) at 4°C overnight and then incubated with a HRP-conjugated goat anti-rabbit IgG antibody (1 : 5000, AS014, ABclonal, China) for 2 h. The protein levels were normalized to that of GAPDH (1 : 5000, AC002, ABclonal, China) to control for the loading efficiency.

The immunoblots were visualized using chemiluminescence and were analyzed using a Bio-Imaging System (Syngene, Cambridge, UK). The relative density of each band is expressed in arbitrary density units after subtracting the background.

### 2.10. Transient Transfection and Luciferase Assays

Online databases (http://www.microrna.org/; http://mirdb.org/index.html) were used to predict the putative binding site of miR-873 in 3′-UTR of ABCA1. U251 cells were transiently cotransfected with a luciferase reporter plasmid encoding the 3′-untranslated region (3′-UTR) of ABCA1 (pMIR-Report-ABCA1) and the pcDNA-3.1(-)-miR-873 vector or an empty vector. A dual-luciferase reporter assay system was used to detect the functional regulation of ABCA1 mRNA by miR-873. The luciferase activity was quantified 36 h after transfection using the Dual- Luciferase Reporter Gene Assay Kit (Promega, Madison, WI, USA) and a luminometer system (Tecan Spark™ 10M, Tecan, Switzerland). The firefly luciferase activity was normalized with the Renilla luciferase activity to control for the transfection efficiency.

### 2.11. SH-SY5Y Cell Line Overexpressing *α*-Synuclein

SH-SY5Y cells were transfected with the pHAGE-CMV-MCS-IZsGreen vector containing human *α*-synuclein cDNA using the Lipofectamine™ 2000 transfection reagent (Invitrogen, Carlsbad, CA, USA). After transfection for 48 h, the cells were reseeded at low cell density in DMEM containing 8 *μ*g/ml puromycin. Puromycin was used for two weeks, and the surviving clones were isolated and transferred to new plates until they had grown into a colony. The expression level of eGFP was determined to evaluate the transfection efficiency.

### 2.12. Fluorescence Immunocytochemistry

To investigate the effects of miR-873 on the lysosomal levels of cholesterol and *α*-synuclein, normal SH-SY5Y cells or SH-SY5Y cells overexpressing *α*-synuclein were transfected with the miR-873 expression vector or an empty vector. After transfection for 48 h, the cells were fixed with 4% paraformaldehyde and coincubated with a polyclonal rabbit anti-human LAMP2 antibody (1 : 200, Cat. 10397-1-AP; Proteintech, Wuhan, China) and filipin (125 mg/ml) or a monoclonal mouse anti-human *α*-synuclein antibody (1 : 400, Cat. ab1903; Abcam, Cambridge MA, USA). The secondary antibodies included a FITC-conjugated goat anti-mouse IgG antibody (1 : 100, Kerui Biotechnology, Wuhan, China) and a TRITC-conjugated goat anti-rabbit IgG antibody (1 : 100, Kerui Biotechnology). Images were obtained with a laser confocal scanning microscope (Leica, Wetzlar, Germany).

To investigate the effects of miR-873 on the autophagic flux, SH-SY5Y cells were cotransfected with an enhanced green fluorescent protein- (pEGFP-) LC3 plasmid (Cat. #24920; Addgene, Cambridge, MA, USA) and miR-873 or the empty vector. After transfection for 6 h, the cells were treated with rapamycin (200 nM). To investigate the effects of ABCA1 on the autophagic flux, SH-SY5Y cells were cotransfected with the pEGFP-LC3 plasmid and sh-ABCA1. The cells were fixed and the nuclei were stained with 4-6-diamidino-2-phenylindole (DAPI, Promoter Biotechnology). Images were obtained with a confocal fluorescence microscope (Leica LCS SP8 STED, German).

### 2.13. Statistical Analysis

The normalized mRNA levels of miR-873, pre-miR-873, ABCA1, A20, GCase, and CTSD are expressed as arbitrary density units. The levels of the ABCA1 protein normalized to *β*-actin and autophagy-related proteins normalized to GAPDH are expressed as arbitrary density units. The mean fluorescence intensity and colocalization coefficient were analyzed using ImageJ, and are expressed as arbitrary density units. Identical illumination and camera settings were used within each data set. For the in vivo study, the data are expressed as the arithmetic mean and standard deviation (mean ± S.D.). The in vitro data were obtained from at least three separate experiments, and are expressed as the arithmetic mean and standard error of the mean (mean ± S.E.M.). The differences among the groups were tested by one-way ANOVA followed by the least significant difference (LSD) post hoc test. A value of *p* < 0.05 was considered significant.

## 3. Results

### 3.1. The Inhibition of LPS-Induced Damage to DA Neurons by the miR-873 Sponge in Rats

**Figure 1:**
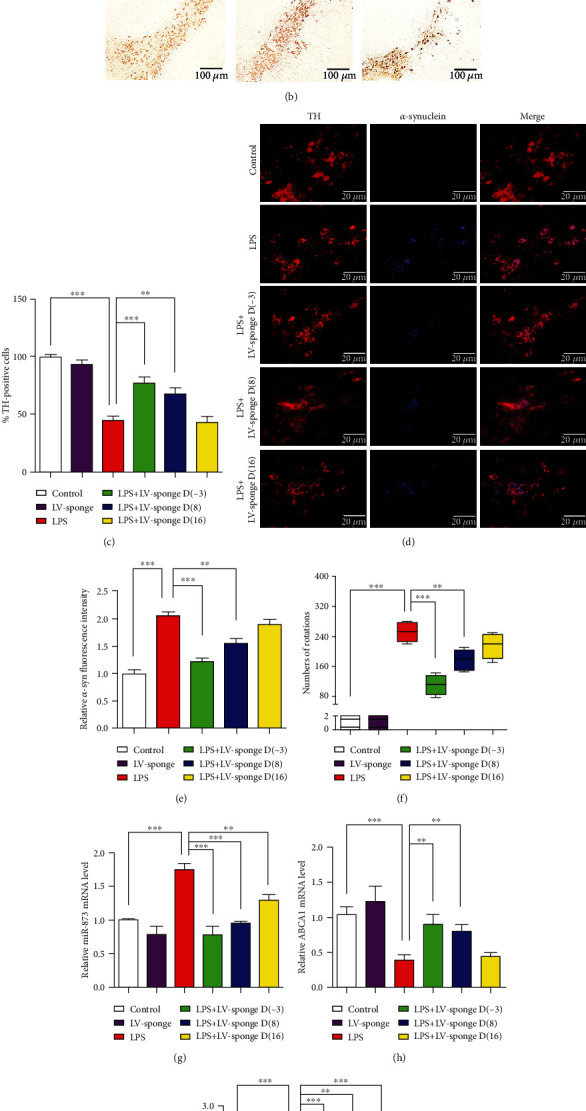
The effects of the miR-873 inhibitor on the damage to DA neurons in the substantia nigra pars compacta in a LPS-induced rat model of PD. The animals were transfected with the miR-873 sponge 3 days before LPS treatment or 8 and 16 days after LPS treatment (a). The damage to DA neurons following LPS treatment was detected by immunohistochemistry staining (*n* = 5) (b). The reduction in the tyrosine hydroxylase- (TH-) positive cells on the lesioned side was attenuated in the rats transfected with the miR-873 sponge 3 days before LPS treatment or 8 days after LPS injection, compared with LPS treatment alone (c). The accumulation of *α*-synuclein in DA neurons was examined by fluorescence immunohistochemistry (*n* = 5) (d and e). The number of apomorphine-induced rotations following LPS treatment was decreased in the rats treated with the miR-873 sponge compared with the rats treated with LPS alone (*n* = 10) (f). The mRNA levels of miR-873 were increased by LPS treatment, compared with the control (*n* = 5) (g). Transfection of the miR-873 sponge attenuated the inhibition of the mRNA levels of ABCA1 (h) and A20 (i) following LPS treatment. The data are expressed as the mean ± S.D.; ^∗^*p* < 0.05, ^∗∗^*p* < 0.01, and ^∗∗∗^*p* < 0.001 compared with the controls.

To investigate the effects of the miR-873 sponge on the damage to DA neurons induced by LPS, the rats received stereotactic injections of the miR-873 sponge 3 days before LPS treatment or 8 and 16 days after LPS treatment (Figure 1(a)). On day 22 after LPS treatment, the DA neurons on the lesioned SN side were reduced by 55.5% (*p* < 0.001) compared with those on the intact side; however, the DA neurons were decreased by 22.8% (*p* < 0.001) in the rats injected with the miR-873 sponge 3 days before LPS treatment, and by 32.8% (*p* < 0.01) in the rats injected with the miR-873 sponge 8 days after LPS treatment (Figures 1(b) and 1(c)). No significant change in the DA neurons remaining on the lesioned SN side was observed in the rats injected with the miR-873 sponge 16 days after LPS treatment, compared with those in the rats injected with LPS alone. The obvious accumulation of the *α*-synuclein protein (blue) in DA neurons (red) was observed 22 days after LPS treatment, while the *α*-synuclein protein was slightly increased in the rats treated with the miR-873 sponge 3 days before LPS treatment (Figures 1(d) and 1(e)). In the LPS group, the average number of rotations within 30 min after apomorphine injection was 248. Compared with those of the rats treated with LPS alone, the rotations of the rats treated with the miR-873 sponge 3 days before LPS treatment were reduced by 60.5% (*p* < 0.001), and the rotations of the rats treated with the miR-873 sponge 8 days after LPS treatment were reduced by 33.5% (*p* < 0.01) (Figure 1(f)). A slight decrease in rotations was observed in the rats treated with the miR-873 sponge 16 days after LPS treatment compared with those in the rats treated with LPS alone (Figure 1(f)). The data suggest that the miR-873 sponge can effectively improve the damage to DA neurons in the LPS-induced model of PD.

Compared with the control treatment, the LPS treatment significantly increased the miR-873 mRNA levels and decreased the ABCA1 mRNA levels (Figures 1(g) and 1(h)). A previous study showed that miR-873 regulated the A20 levels in mouse primary astrocytes [13]. Compared with the LPS treatment alone, the injection of the miR-873 sponge 3 days before LPS treatment increased the ABCA1 mRNA levels by 132% (*p* < 0.01), and the injection of the miR-873 sponge 8 days after LPS treatment increased the ABCA1 mRNA levels by 104% (*p* < 0.01) (Figure 1(h)); in addition, the A20 levels were increased by 193% (*p* < 0.001) when the miR-873 sponge was injected 3 days before LPS treatment, and by 149% (*p* < 0.01) when the miR-873 sponge was injected 8 days after LPS treatment. The A20 mRNA levels were increased when the miR-873 sponge was injected 16 days after LPS treatment compared with LPS treatment alone (Figure 1(i)); however, no change in the ABCA1 mRNA levels was observed (Figure 1(h)). The data suggest that the miR-873 sponge can attenuate the LPS-induced inhibition of ABCA1 and A20.

### 3.2. Involvement of the TLR4-MyD88 Signaling Pathway in the Regulation of the miR-873 and ABCA1 Levels by LPS in U251 Cells

**Figure 2:**
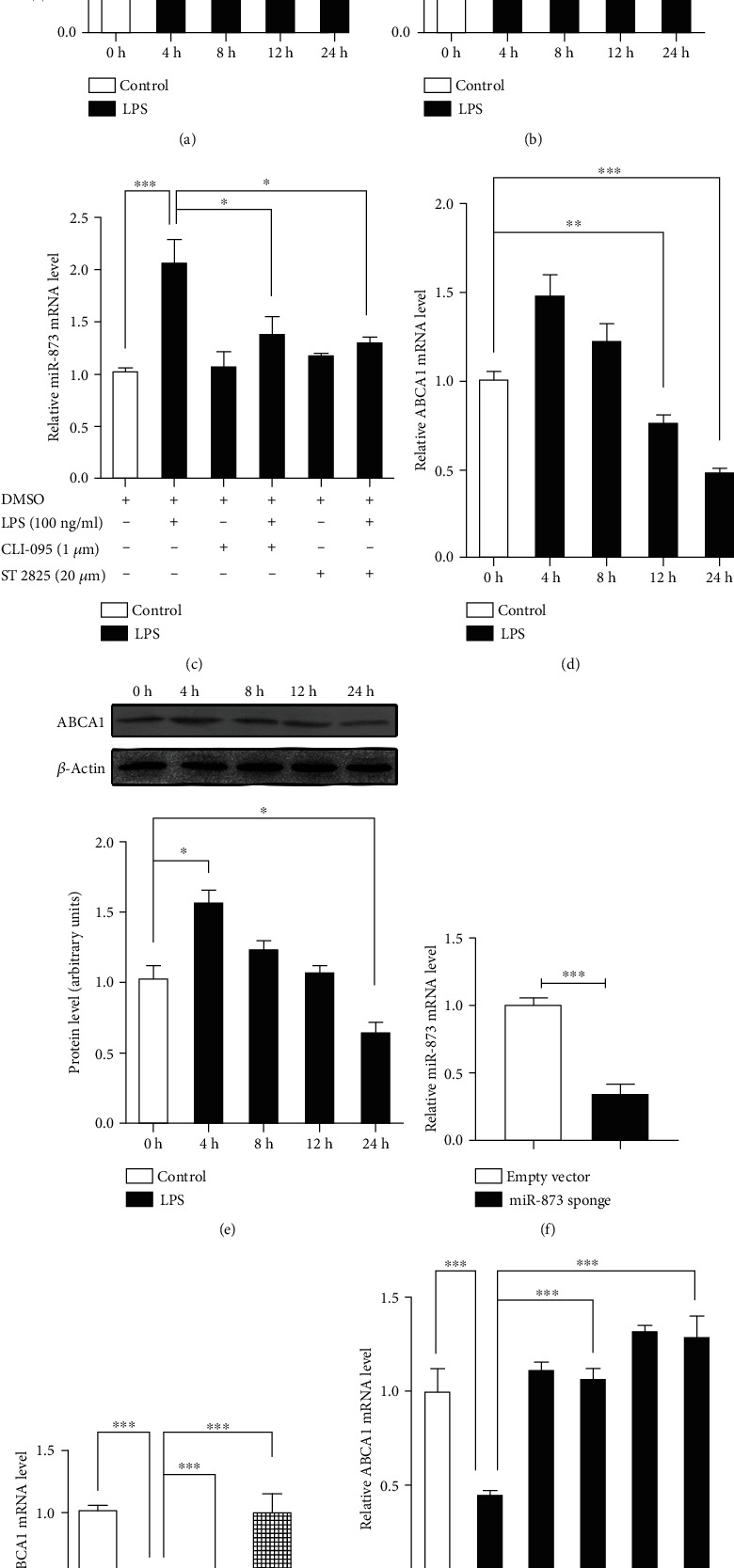
The downregulation of ABCA1 by LPS via miR-873 in human glioblastoma U251 cells. The pre-miR-873 mRNA level was increased 4 h to 8 h after LPS treatment and returned to the control levels by 12 h (a); additionally, the miR-873 mRNA level was increased after LPS treatment for 12 h (b). The induction of miR-873 following LPS treatment for 24 h was eliminated by a TLR4 inhibitor (CLI-095) and a MyD88 inhibitor (ST2825) (c). The mRNA level of ABCA1 was increased 4 h after LPS treatment, but a significant decrease in ABCA1 was observed from 12 h to 24 h (d). The protein level of ABCA1 was reduced after LPS treatment for 24 h (e). Transfection of the miR-873 sponge decreased the mRNA levels of miR-873 (f). Transfection of the miR-873 sponge eliminated the increase in the ABCA1 mRNA levels 24 h after LPS treatment (g). The reduction in the ABCA1 levels was eliminated by a TLR4 inhibitor (CLI-095) and a MyD88 inhibitor (ST2825) following LPS treatment for 24 h (H). The data are expressed as the mean ± S.E.M.; *n* = 3, ^∗^*p* < 0.05, ^∗∗^*p* < 0.01, and ^∗∗∗^*p* < 0.001 compared with the respective controls.

Compared with the controls, the pre-miR-873 mRNA level was markedly increased after LPS treatment for 4 h to 8 h; meanwhile, the miR-873 mRNA level was increased after LPS treatment for 12 h to 24 h (Figures 2(a) and 2(b)). The miR-873 mRNA level was significantly increased by 106% (*p* < 0.01) after LPS treatment for 24 h compared with the controls; however, the specific inhibitors of TLR4 (CLI095) and MyD88 (ST2825) attenuated the LPS-induced upregulation of miR-873 (Figure 2(c)). The data indicated the involvement of the TLR4-MyD88 signaling pathway in the regulation of miR-873 by LPS.

The ABCA1 mRNA levels were increased by 47% (*p* < 0.01) at 4 h but decreased by 24.5% (*p* < 0.01) at 12 h after LPS treatment compared with the controls (Figure 2(d)); additionally, the ABCA1 protein level was decreased by 36.5% (*p* < 0.05) 24 h following LPS treatment (Figure 2(e)). Cytokines including IL-1*β* and TNF*α* decreased the ABCA1 mRNA levels in SH-SY5Y cells (Figures S2 and S3). However, no changes in the cholesterol transporters NPC1 and NPC2 were observed following exposure to inflammatory stimuli, including LPS, IL-1*β*, and TNF*α* (Figures S1–S3).

The decrease in ABCA1 (55.3%, *p* < 0.01) after LPS treatment for 24 h was eliminated by both CLI095 and ST2825 (Figure 2(h)). A previous study showed that ABCA1 was a downstream target gene of liver X receptors (LXRs) [24]. However, the LXR*α* mRNA level was increased 4 h after LPS treatment and returned to the control level 8 h after LPS treatment (data not shown). The data suggest that LXR*α* may contribute to the upregulation of ABCA1 at the early stage of LPS treatment, but not to the downregulation of ABCA1.

Transfection of the miR-873 sponge significantly decreased the mRNA levels of miR-873 (Figure 2(f)). The transfection of miR-873 exacerbated the decreases in the ABCA1 mRNA levels after LPS treatment for 24 h, but the miR-873 sponge completely eliminated the inhibition of ABCA1 by LPS (Figure 2(g)). The data indicate that miR-873 may contribute to the long-term effects of LPS treatment on ABCA1.

### 3.3. ABCA1 Was Downregulated by miR-873

**Figure 3:**
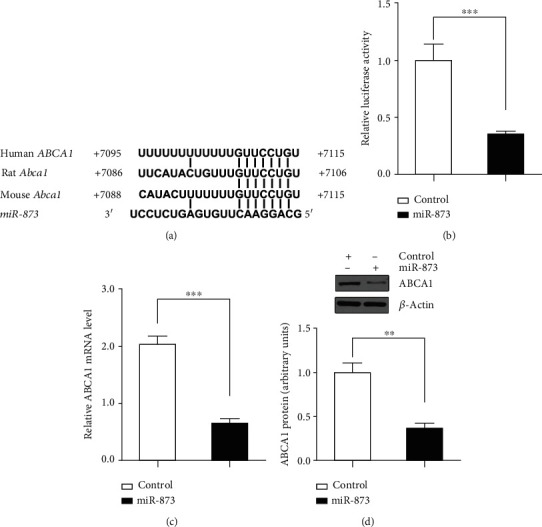
The regulation of ABCA1 expression by miR-873 in U251 cells. The luciferase activity in the cells transfected with the plasmid expressing the ABCA1 3′-untranslated region (3′-UTR) reporter was inhibited following cotransfection of the miR-873 expression vector (b). The predicted binding sites of miR-873 in the 3′-UTR of the human ABCA1 gene and the rodent Abca1 gene are shown (a). The mRNA (c) and protein (d) levels of ABCA1 were decreased following transfection with miR-873. The basal activity levels measured in the cells transfected with the empty vector were set to 1. The data are expressed as the mean ± S.E.M.; *n* = 3, ^∗∗^*p* < 0.01 and ^∗∗∗^*p* < 0.001 compared with the respective controls.

The luciferase activity of the ABCA1 3′-UTR reporters was inhibited by miR-873 by 64.7% (*p* < 0.001) in U251 cells (Figures 3(a) and 3(b)). Consistent with the 3′-UTR reporter results, the ABCA1 mRNA level was decreased by 68% (*p* < 0.001) after the transfection of the miR-873 expression vector compared with the transfection of the empty vector (Figure 3(c)). In addition, the ABCA1 protein level was decreased by 63.5% (*p* < 0.01) by miR-873 transfection (Figure 3(d)). The data suggest that the binding sites of miR-873 are functional.

### 3.4. Effects of miR-873 on the Lysosomal Cholesterol and *α*-Synuclein Levels in SH-SY5Y Cells

**Figure 4:**
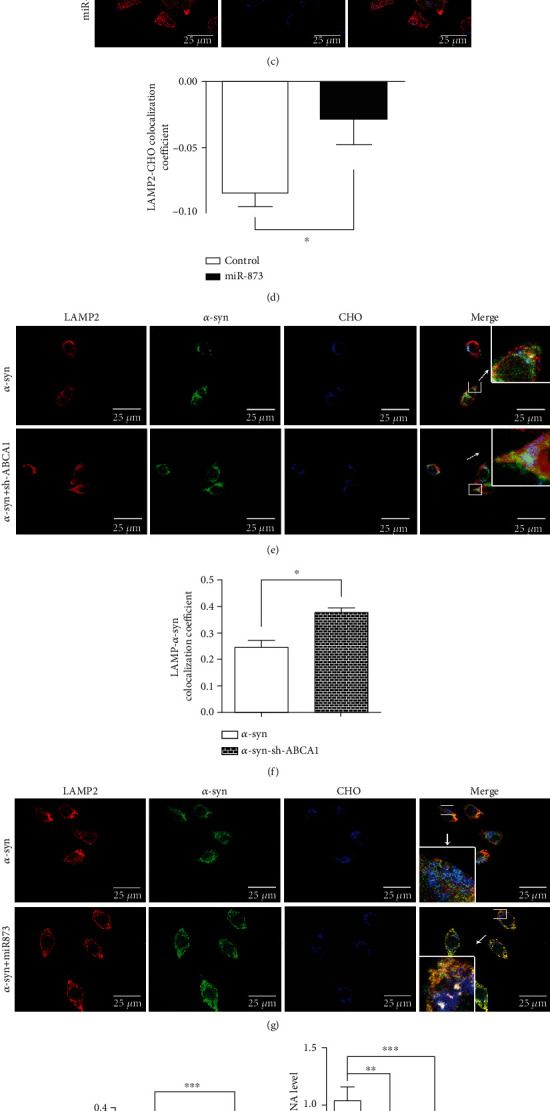
The effects of ABCA1 silencing or miR-873 transfection on the levels of free cholesterol and *α*-synuclein in the lysosomes of normal SH-SY5Y cells or SH-SY5Y cells overexpressing *α*-synuclein. The levels of free cholesterol labeled with filipin (blue) in the lysosomes labeled with anti-LAMP-2 antibody (red) were increased in SH-SY5Y cells following ABCA1 silencing, as indicated by the colocalization coefficient (a and b). The lysosomal cholesterol was increased in SH-SY5Y cells following miR-873 transfection, as indicated by the colocalization coefficient (c and d). The distribution of *α*-synuclein (green) in the lysosomes labeled with anti-LAMP-2 antibody (red) was increased following ABCA1 silencing in SH-SY5Y cells overexpressing *α*-synuclein, as indicated by the colocalization coefficient (e and f). The levels of *α*-synuclein in the lysosomes were increased following miR-873 transfection in SH-SY5Y cells overexpressing *α*-synuclein, as indicated by the colocalization coefficient (g and h). The transfection of miR-873 reduced the mRNA levels of cathepsin D (CTSD) (i) and GCase (j) in SH-SY5Y cells overexpressing *α*-synuclein. The model of the disruption of intracellular cholesterol trafficking by miR-873 via ABCA1 in neuronal cells is shown (k). The data are expressed as the mean ± S.E.M.; *n* = 3, ^∗^*p* < 0.05, ^∗∗^*p* < 0.01, and ^∗∗∗^*p* < 0.001 compared with the controls.

The fluorescence immunocytochemistry assay showed that the free cholesterol (blue) levels in the lysosomes (labeled with LAMP2 staining (red)) were altered following ABCA1 silencing (Figure 4(a)). The colocalization coefficient of cholesterol and the LAMP2 protein indicated an increase in the lysosomal cholesterol levels after sh-ABCA1 transfection compared with the controls (Figure 4(b)). In addition, the lysosomal cholesterol levels were increased following miR-873 transfection, as indicated by the colocalization coefficient (Figures 4(c) and 4(d)).

In SH-SY5Y cells overexpressing *α*-synuclein, the colocalization coefficient of *α*-synuclein (green) and the LAMP2 protein (red) indicated an increase in the *α*-synuclein levels in the lysosome following ABCA1 silencing, compared with the controls (Figures 4(e) and 4(f)). miR-873 transfection altered the intracellular distribution of *α*-synuclein. The colocalization coefficient of *α*-synuclein and the LAMP2 protein indicated an increase in the *α*-synuclein levels in the lysosomes, compared with the controls (Figures 4(g) and 4(h)).

Extensive accumulation of endogenous *α*-synuclein in neurons was observed in a mouse model of CTSD deficiency [25]. The reduced activity of GCase, which is a nonprotein-degrading lysosomal enzyme, was also reported to increase the accumulation of *α*-synuclein [26]. Compared with the controls, the levels of CTSD and GCase were decreased in SH-SY5Y cells overexpressing *α*-synuclein (Figures 4(i) and 4(j)). The data were consistent with previous reports that *α*-synuclein inhibited the activity of GCase [27, 28]. miR-873 transfection inhibited the mRNA levels of CTSD and GCase in SH-SY5Y cells (Figure S5). In SH-SY5Y cells overexpressing *α*-synuclein, miR-873 exacerbated the *α*-synuclein-induced inhibition of both the CTSD and GCase mRNA levels. The data suggest that miR-873 may exacerbate *α*-synuclein-induced lysosomal dysfunction.

### 3.5. Effects of miR-873 on Autophagy in SH-SY5Y Cells

**Figure 5:**
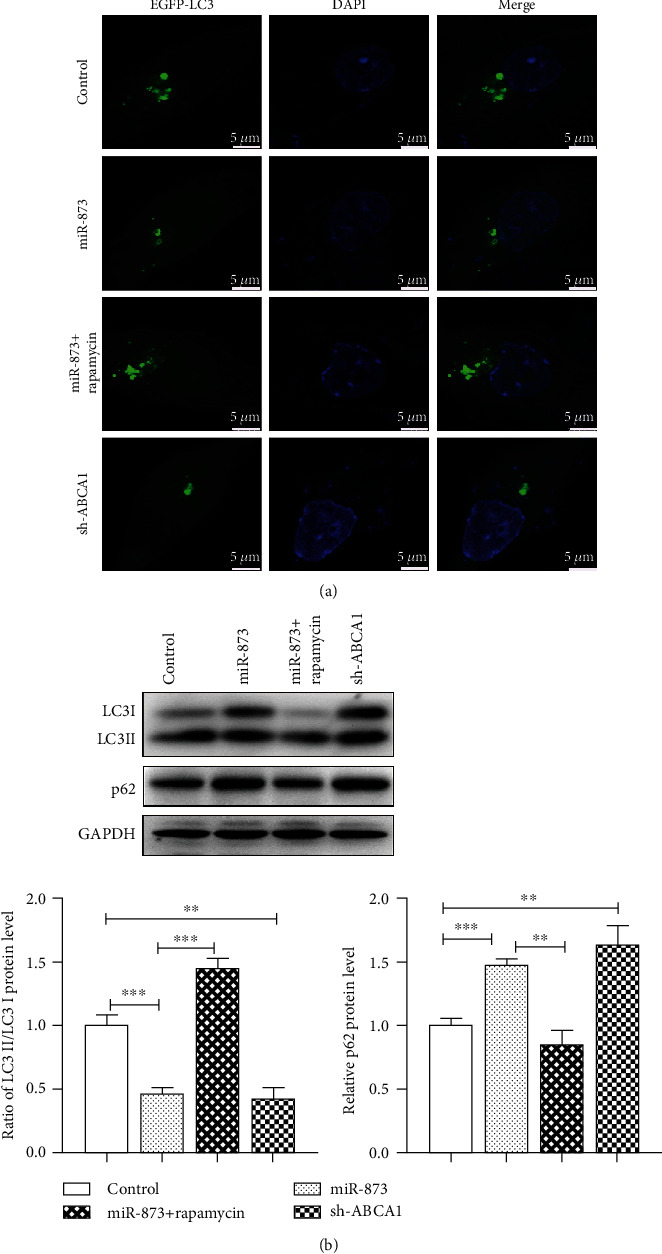
The effects of miR-873 on autophagy in SH-SY5Y cells. The fluorescence of pEGFP-LC3 (green) was reduced in the cytoplasm of the cells (the nucleus was stained blue) transfected with miR-873 or sh-ABCA1 (a). The allosteric inhibitor of mTORC1, rapamycin, restored the miR-873-induced reduction in autophagic flux. The LC3II/LC3I protein expression ratio was reduced in the cells transfected with miR-873 or sh-ABCA1; moreover, the p62 protein accumulated in these cells (b). The data are expressed as the mean ± S.E.M.; *n* = 3, ^∗^*p* < 0.05, ^∗∗^*p* < 0.01, and ^∗∗∗^*p* < 0.001 compared with the controls.

As the main mechanism of *α*-synuclein clearance is lysosome-dependent autophagy, we investigated the effect of miR-873 on autophagy. The autophagic flux was evaluated by confocal microscopy after the transfection with the pEGFP-LC3 vector. The fluorescence of pEGFP-LC3 (green) was significantly reduced in the cytoplasm of the cells (the nucleus was stained blue) transfected with miR-873 or sh-ABCA1 (Figure 5(a)). The data suggest that the inhibition of the autophagic flux caused by miR-873 transfection may be due to the inhibition of ABCA1. Rapamycin, which is an allosteric inhibitor of mTORC1, restored the miR-873-induced reduction in the autophagic flux.

The LC3II/LC3I protein expression ratio was significantly reduced in the cells transfected with miR-873 or sh-ABCA1 compared with the controls; in addition, accumulation of the p62 protein was observed in these cells (Figure 5(b)). Rapamycin completely eliminated the accumulation of the p62 protein and the reduction in the LC3II/LC3I protein expression ratio in the cells transfected with miR-873 or sh-ABCA1. The data suggest that miR-873 inhibits autophagy.

### 3.6. Effects of Silencing ABCA1 or A20 on LPS-Induced Damage to DA Neurons in Rats

**Figure 6:**
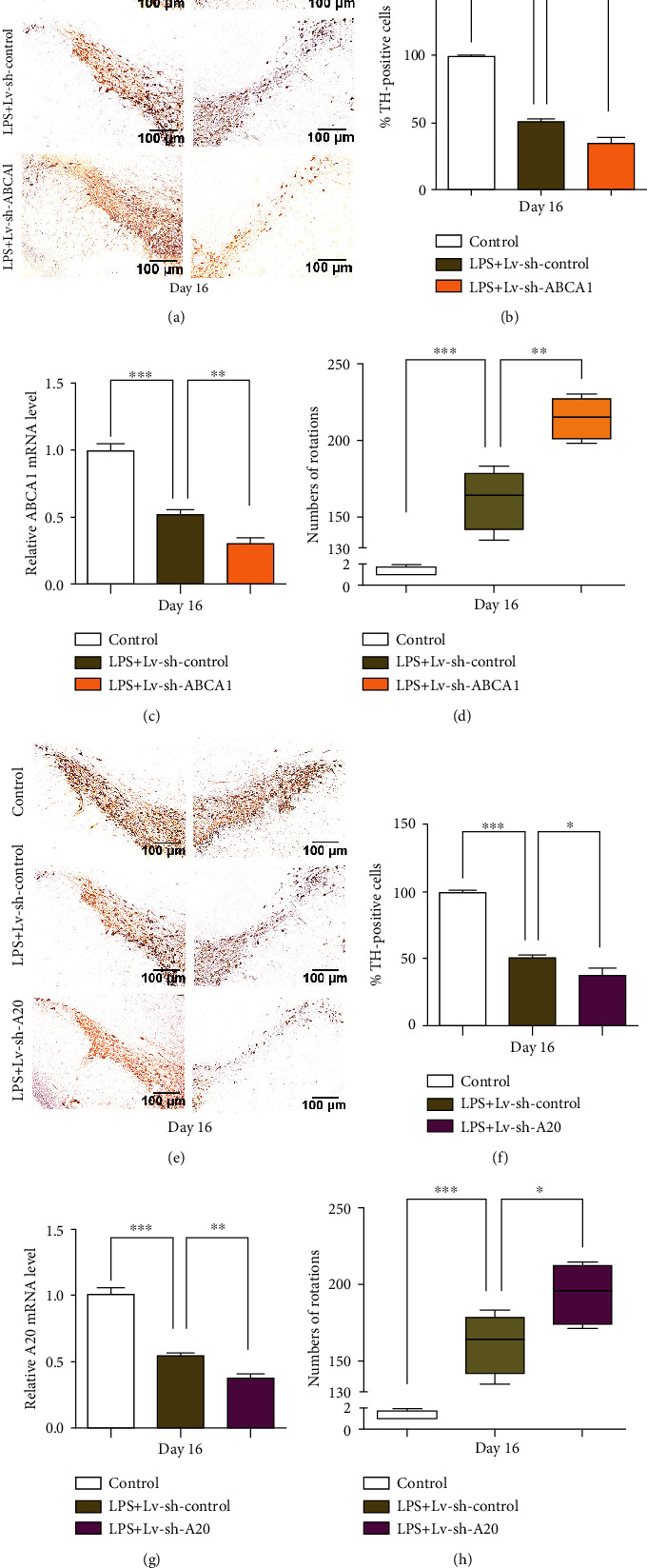
The effects of sh-ABCA1 or sh-A20 transfection on DA neuron damage in a LPS-induced rat model of PD. The LPS-induced loss of DA neurons in the substantia nigra pars compacta of rats with ABCA1 (a) or A20 (e) knockdown was detected by immunohistochemistry staining (*n* = 5). The percentage of tyrosine hydroxylase- (TH)- positive cells on the lesioned side relative to those on the intact side of rats with ABCA1 (b) or A20 (f) knockdown was calculated by ImageJ. The alteration of the mRNA levels of ABCA1 (c) and A20 (g) following LPS treatment (*n* = 5). The number of rotations in the rats with ABCA1 (d) or A20 (h) knockdown 16 days after LPS injection (*n* = 10). The data are expressed as the mean ± S.D.; ^∗^*p* < 0.05, ^∗∗^*p* < 0.01, and ^∗∗∗^*p* < 0.001 compared with the controls.

The DA neurons were labeled by tyrosine hydroxylase using immunohistochemistry. The number of DA neurons was reduced by 49.8% (*p* < 0.001) on the lesioned SN side compared with that on the intact side 16 days after stereotactic injection with LPS (Figures 6(a) and 6(b)). Compared with LPS+sh-control group, the loss of DA neurons was increased by 31.9% (*p* < 0.05) by ABCA1 silencing; additionally, the number of rotations induced by apomorphine was increased by 30.5% in the rats with ABCA1 knockdown 16 days after LPS injection (*p* < 0.01) (Figure 6(d)).

We confirmed that miR-873 could target A20 and affect the NF-*κ*B signaling pathway in human astrocytes (Figure S6). To investigate the effects of A20 on LPS-induced damage to DA neurons, rats received LV-sh-A20 injection before LPS treatment. Compared with the intact side, the number of DA neurons on the lesioned SN side was reduced by 49.8% (*p* < 0.001) 16 days after LPS injection. Compared with the LPS+sh-control group, the loss of DA neurons was increased by 25.3% (*p* < 0.05) by LPS in the rats with A20 knockdown (Figures 6(e) and 6(f)); moreover, the number of rotations induced by apomorphine was increased by 20.1% (*p* < 0.05) in the rats with A20 knockdown 16 days after LPS injection (Figure 6(h)).

## 4. Discussion and Conclusions

This is the first demonstration that the inhibition of miR-873 efficiently improved the damage to DA neurons induced by neuroinflammation. We showed that (i) the miR-873 sponge significantly ameliorated the LPS-induced accumulation of *α*-synuclein and loss of DA neurons in the SN; (ii) miR-873 was a key mediator of the inhibition of ABCA1 by LPS through the TLR4-MyD88 signaling pathway; (iii) miR-873 transfection or ABCA1 silencing resulted in the accumulation of *α*-synuclein in lysosomes and the impairment of autophagy; and (iv) ABCA1 and A20 silencing exacerbated the LPS-induced damage to DA neurons. These data indicated that the inhibition of miR-873 can potentially prevent the damage to DA neurons caused by neuroinflammation.

In the present study, we demonstrated that miR-873 suppressed ABCA1 expression by binding to the 3′-UTR of ABCA1 mRNA. The ABCA1 protein localizes to the cell surface and to the late endosomes/lysosomes and is responsible for the intracellular cholesterol homeostasis [29]. Our data showed that ABCA1 silencing or miR-873 transfection induced cholesterol accumulation in lysosomes and impaired autophagy. A previous study showed that the excess ox-LDL inhibited ABCA1 expression, leading to the inhibition of autophagy [30]. Autophagy-mediated cholesterol efflux in macrophage foam cells is primarily dependent on ABCA1, which itself is linked to the endosomal/lysosomal cholesterol pools [31]. A recent study showed that lysosomal cholesterol activated mTORC1 [32], which can cluster on LAMP2-positive lysosomes and function as a downstream molecule for nutrient sensing. Our data showed that rapamycin restored the miR-873-induced inhibition of the autophagic flux, suggesting that rapamycin could re-drive the autophagic signaling pathway via the inhibition of mTORC1.

ABCA1 expression was inhibited in both human astrocytes and rat SN regions during chronic neuroinflammation. The ABCA1 protein may function as a mediator of the cross-talk between cholesterol homeostasis and typical inflammatory responses in neuronal cells. The TLR4-MyD88 signaling pathway was involved in the upregulation of miR-873 and the downregulation of ABCA1 following LPS treatment. A recent study has shown that polymorphisms in the TLR4 gene are associated with sporadic PD and early-onset PD [33]. miR-873 transfection or ABCA1 silencing induced the accumulation of *α*-synuclein in lysosomes, suggesting that alteration of the intracellular cholesterol transporter can impair the clearance of *α*-synuclein. A previous study showed that the LPS-induced levels of NF-*κ*B and TNF*α* were increased in macrophages with ABCA1 silencing [34]. The upregulation of ABCA1 by LPS from *Salmonella minnesota* R595 was observed in THP-1 cells [35]. The inconsistent pattern of ABCA1 regulation by LPS may be due to differences in the LPS sources or cell lines. A previous report showed that the TLRs/NF-*κ*B signaling pathway was activated in U251 cells by LPS from *Escherichia coli* (*E. coli*), but not efficiently initiated by LPS from *Salmonella minnesota* R595 [36]. The downregulation of ABCA1 by LPS from *E. coli* was observed in RAW 264.7 cells [37].

Our data showed that A20 knockdown exacerbated the damage to DA neurons in the LPS-induced model of PD. A20 can inhibit the E3 ligase activities of TRAF6 and inhibit NF-*κ*B signaling [38]. The overexpression of miR-873 remarkably promoted the activation of NF-*κ*B and induced the production of inflammatory cytokines and chemokines [13].

Taken together, these results showed that chronic neuroinflammation inhibited the expression of ABCA1 via the upregulation of miR-873. ABCA1 silencing or miR-873 transfection resulted in the impairment of autophagy and *α*-synuclein accumulation. The upregulation of miR-873 could also aggravate the NF-*κ*B signaling pathway via the inhibition of A20. The miR-873 sponge could effectively relieve the damage to DA neurons caused by neuroinflammation, suggesting that the miR-873 inhibitor may play a dual protective role in PD by improving intracellular cholesterol homeostasis and neuroinflammation.

.

## Data Availability

The figure and table data used to support the findings of this study are included within the article.

## Abbreviations

PD: Parkinson's disease
ABCA1: ATP-binding cassette subfamily A member 1
LPS: Lipopolysaccharide
LV: Lentiviral vector
A20: TNF alpha-induced protein 3
NF-*κ*B: Nuclear factor kappa-B
DA: Dopamine
SN: Substantia nigra
SNpc: Substantia nigra pars compacta
TH: Tyrosine hydroxylase
TLR4: Toll-like receptor 4
MyD88: MYD88 innate immune signal transduction adaptor
SNCA: Synuclein alpha
miRNA: MicroRNA
3′-UTR: 3′-Untranslated region
GCase: Glucosylceramidase beta
CTSD: Cathepsin D
LAMP2: Lysosomal-associated membrane protein 2
DMEM: Dulbecco's modified Eagle medium
shRNA: Short hairpin RNA
PBS: Phosphate-buffered saline
ANOVA: Analysis of variance
LXRs: Liver X receptors.

## Additional Points

*What Is Already Known*. (i) Alterations in cholesterol homeostasis were reported in cell or animal models of PD. (ii) The main mechanism of *α*-synuclein clearance is lysosome-dependent autophagy. *What This Study Adds*. (i) The downregulation of ABCA1 by miR-873 resulted in lysosomal dysfunction and *α*-synuclein accumulation. (ii) The inhibition of miR-873 ameliorated dopaminergic neuron damage in a LPS-induced model of PD. *Clinical Significance*. (i) The inhibition of miR-873 may provide therapeutic benefit in Parkinson's disease.

## Supplementary Materials

Supplementary Materials There are 6 figures and 1 table in the supplementary materials. Figures S1–S3: new data to answer the rationale for experiments according to the comments. The data showed that the transporter ABCA1 was altered under the inflammatory stimuli, but other transporters including NPC1 and NPC2 were not changed. Figure S4: new data to show the effects of shRNAs on target genes to answer the question about the efficiency and the exacerbation of the damage observed in the animal model. Figure S5: new data to answer the question about the regulation of miR-873 on the expression of CTSD and GCase according to the comments. Figure S6: this is the figure moved from the manuscript to the supplement according to the comments. Table S1: the primers and conditions for RT-PCR.
