# Supplementary material for: The Inhibition of miR-873 Provides Therapeutic Benefit in a Lipopolysaccharide-Induced Neuroinflammatory Model of Parkinson's Disease
Source: Oxid Med Cell Longev. 2020 Jul 15;2020:8735249. doi: 10.1155/2020/8735249 (PMC7381950; doi:10.1155/2020/8735249)
Supplement: Supplementary Materials — There are 6 figures and 1 table in the supplementary materials. Figures S1–S3: new data to answer the rationale for experiments according to the comments. The data showed that the transporter ABCA1 was altered under the inflammatory stimuli, but other transporters including NPC1 and NPC2 were not changed. Figure S4: new data to show the effects of shRNAs on target genes to answer the question about the efficiency and the exacerbation of the damage observed in the animal model. Figure S5: new data to answer the question about the regulation of miR-873 on the expression of CTSD and GCase according to the comments. Figure S6: this is the figure moved from the manuscript to the supplement according to the comments. Table S1: the primers and conditions for RT-PCR. [file 8735249.f1.docx]

1. *LPS down-regulates the expression of cholesterol transporters in U251 cells*

The toll-like receptors (TLRs) present in the astrocytes are responsible for the recognition of LPS. To investigate the effects of neuroinflammation on the expression of lysosome cholesterol transporters in the astrocytes, U251 cells were treated with LPS (0.1 μg/ml) or 0.1% DMSO (vehicle) for 24 h. Compared with the control group, the mRNA levels of ABCA1 were decreased by 30% (*p* < 0.001) after LPS treatment; however, no changes of the mRNA levels of NPC1 and NPC2 were observed (Fig. S1).


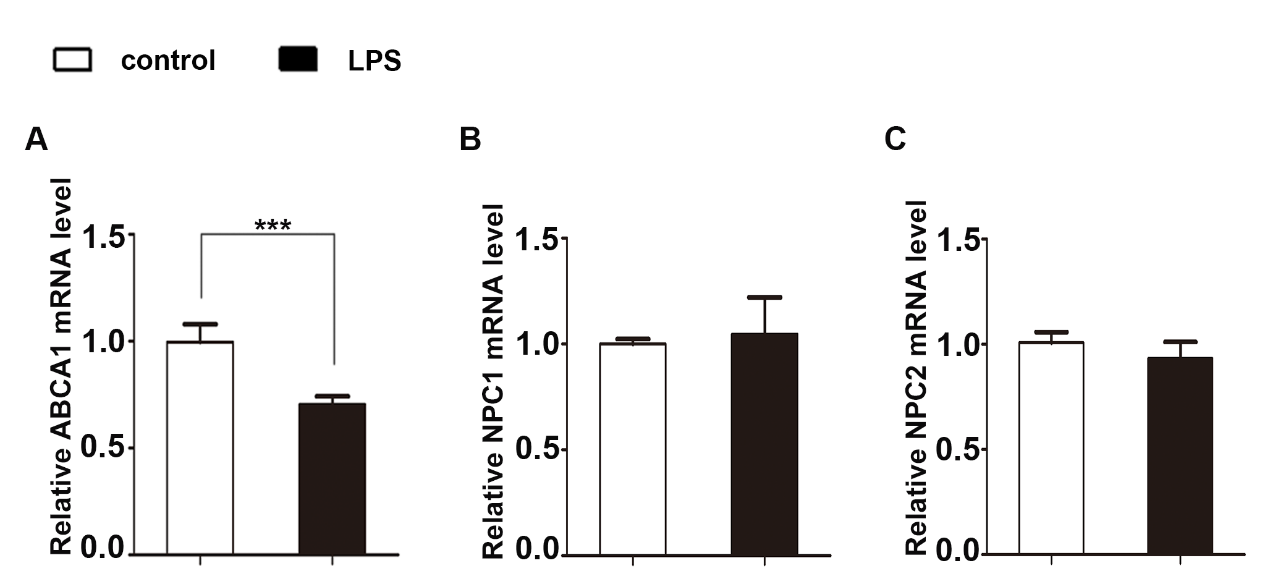


Figure S1 The effects of LPS on the mRNA levels of cholesterol transporters in [human glioblastoma U251 cells](https://www.sciencedirect.com/science/article/pii/S0006899309025104). The data were expressed as mean ± S.E.M.; *n* = 3, * *p* < 0.05, ** *p* < 0.01, *** *p* < 0.001 compared with the respective controls.

1. *IL-1β down-regulates the expression of cholesterol transporters in SH-SY5Y cells*

The cytokine interleukin-1β (IL-1β) is one of the most potent mediators known to play a pivotal role in PD [1, 2]. IL-1β has been found in the CSF and post-mortem striata of PD patients [3-5]. Considering that the proinflammatory molecules released from glial cells are neuron-glia interaction mediators, we investigated the effects of cytokines on the expression of cholesterol transporters in SH-SY5Y cells. SH-SY5Y cells were treated with IL-1β (0.1 μg/ml) or 0.1% DMSO (vehicle) for 24 h. Compared with the control, the ABCA1 mRNA levels were down-regulated by 34.3% (*p* < 0.05) following IL-1β treatment; however, there was no change of the mRNA levels of NPC1 and NPC2 (Fig. S2).


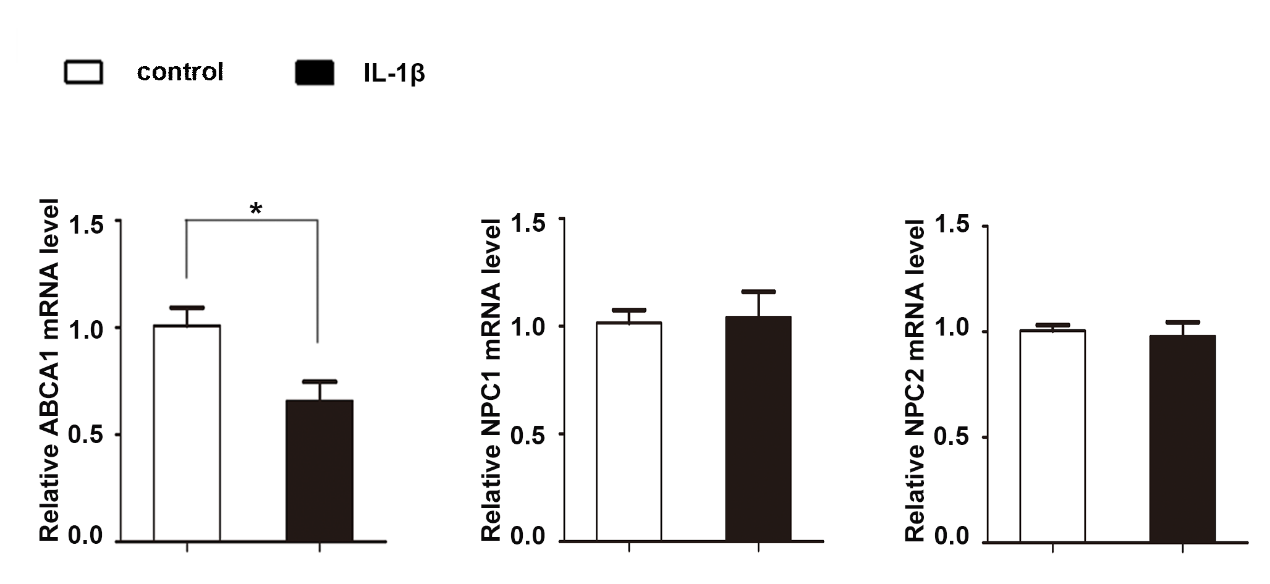


Figure S2 The effects of IL-1β on the mRNA levels of cholesterol transporters in [human neuroblastoma SH-SY5Y cells](https://www.sciencedirect.com/science/article/pii/S0006899309025104). The data were expressed as mean ± S.E.M.; *n* = 3, * *p* < 0.05, ** *p* < 0.01, *** *p* < 0.001 compared with the respective controls.

1. *TNFα down-regulates the expression of cholesterol transporters in SH-SY5Y cells*

Tumor necrosis factor α (TNFα) was induced by the pathological α-synuclein and LPS stimuli, which has been considered to be associated with the pathological process of PD [6, 7]. SH-SY5Y cells were treated with TNFα (0.1 μg/ml) or 0.1% DMSO (vehicle) for 24 h. Compared with the control, the ABCA1 mRNA levels were down-regulated by 47% (*p* < 0.001) following TNFα treatment; however, no changes of the mRNA levels of NPC1 and NPC2 were observed (Fig. S3).


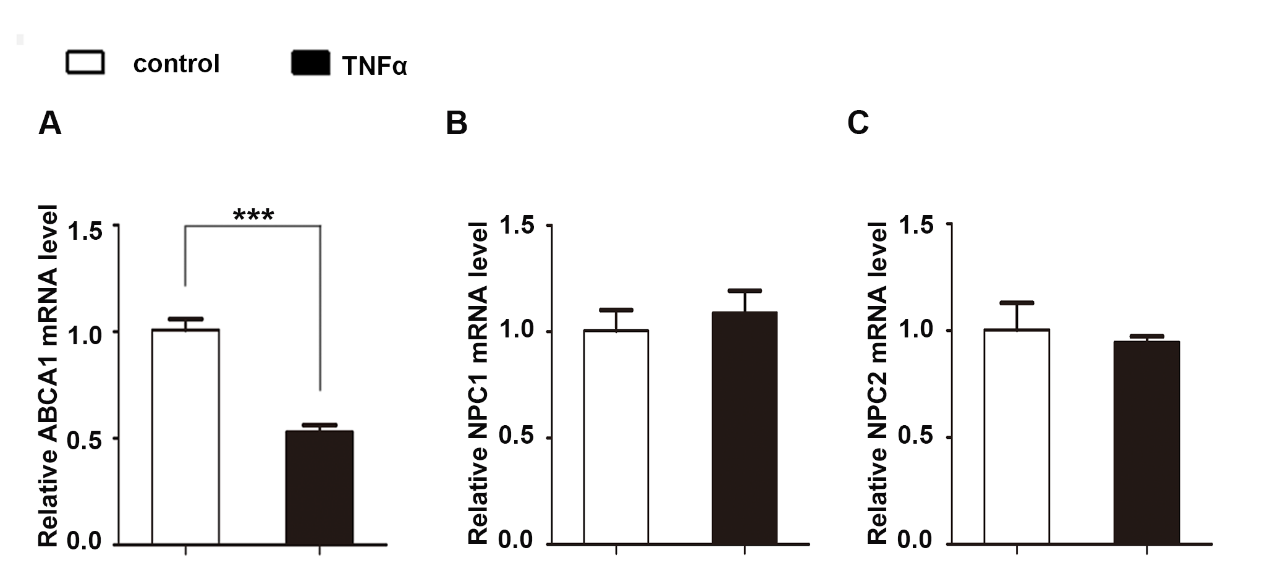


Figure S3 The effects of TNFα on the mRNA levels of cholesterol transporters in [human neuroblastoma SH-SY5Y cells](https://www.sciencedirect.com/science/article/pii/S0006899309025104). The data were expressed as mean ± S.E.M.; *n* = 3, * *p* < 0.05, ** *p* < 0.01, *** *p* < 0.001 compared with the respective controls.

1. *Validation of sh-RNA Silencing Efficiency*

To investigate the silencing efficacy of sh-hABCA1 and sh-rABCA1, human SH-SY5Y cells or rat C6 cells were respectively transfected with the expression vector for 48 h. The mRNA levels of human or rat ABCA1 were significantly decreased in the cells following sh-ABCA1 transfection.

To investigate the silencing efficacy of sh-rA20, rat C6 cells were transfected with the expression vector for 48 h. In C6 cells, the A20 mRNA level was significantly decreased after sh-A20 transfection.


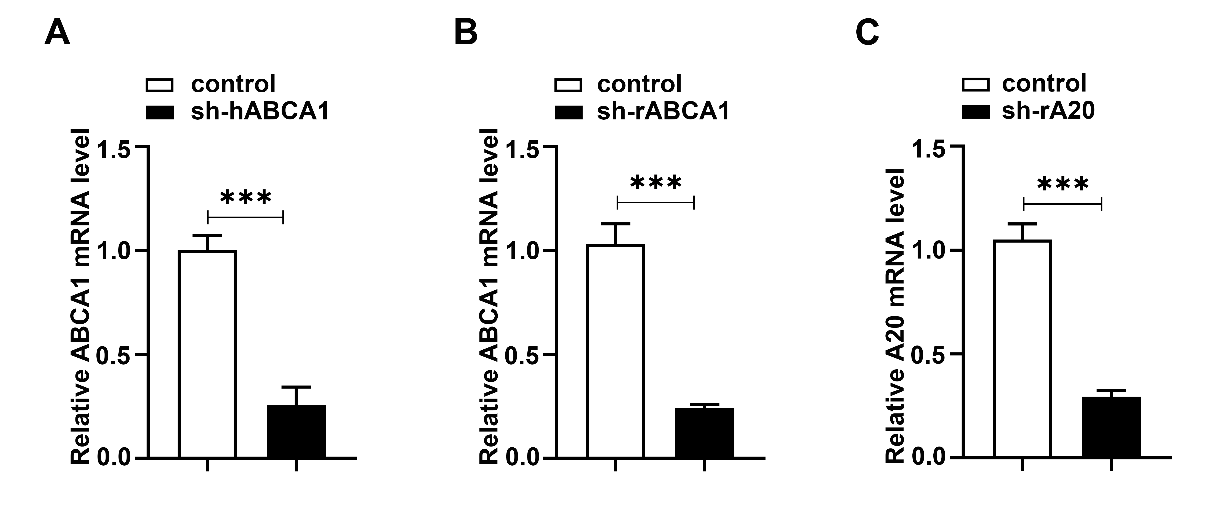


Figure S4 The effects of shRNAs on the mRNA levels of ABCA1 or A20. The inhibition of ABCA1 mRNA by sh-hABCA1 were observed in SH-SY5Y cells (A). The decrease in ABCA1 mRNA by sh-rABCA1 were observed in C6 cells (B). The A20 mRNA level was decreased by sh-rA20 in C6 cells (C). The data were expressed as mean ± S.E.M.; *n* = 3, * *p* < 0.05, ** *p* < 0.01, *** *p* < 0.001 compared with the respective controls.

1. *The effects of miR-873 on CTSD and GCase expression in SH-SY5Y cells*

To investigate the effects of miR-873 on lysosomal enzymes, the SH-SY5Y cells were transfected with miR-873. The mRNA levels of CTSD and GCase were dramatically inhibited by miR-873 in the cells.


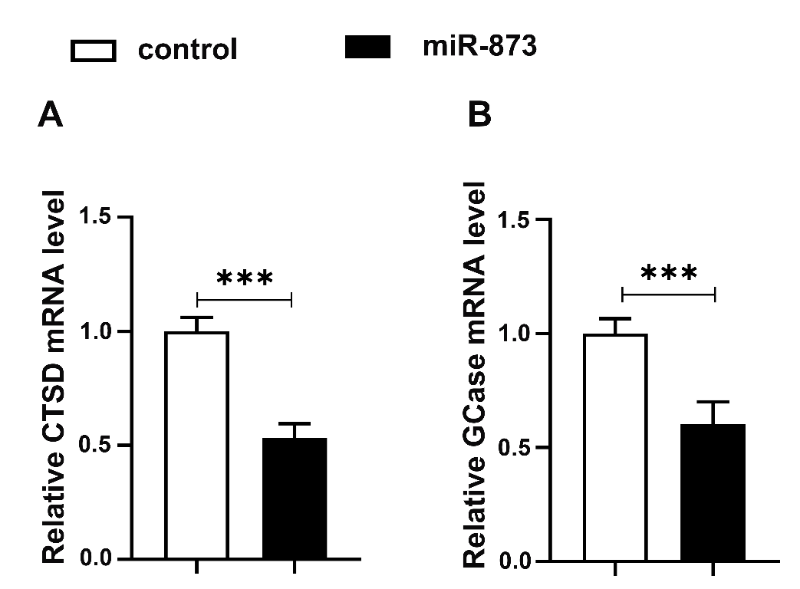


Figure S5 The effects of miR-873 on the mRNA levels of CTSD and GCase. The data were expressed as mean ± S.E.M.; *n* = 3, * *p* < 0.05, ** *p* < 0.01, *** *p* < 0.001 compared with the respective controls.

1. *Activation of NF-kB signalling pathway by miR-873 in U87 cells*

The previous study showed that A20 inhibited NF-κB signalling via the disruption of ubiquitin enzyme complexes [8]. To confirm the effects of miR-873 on NF-κB signalling pathway in human astrocytes, the translocation of p65 (the NF-κB subunit) was assayed following miR-873 transfection for 48 h. Compared with the control, the p65 protein levels in the nucleus from U87 cells were induced following miR-873 transfection, but decreased by miR-873 sponge (Figure S6A). Compared with the controls, the A20 mRNA level was increased at 8 h following LPS treatment, but decreased from 12 h till 24 h by LPS (Figure S6B). Consistently, the A20 mRNA level was decreased by 73.1% after miR-873 transfection (*p* < 0.001), but increased by 133% (*p* <0.05) after miR-873 sponge transfection (Figure S6C).


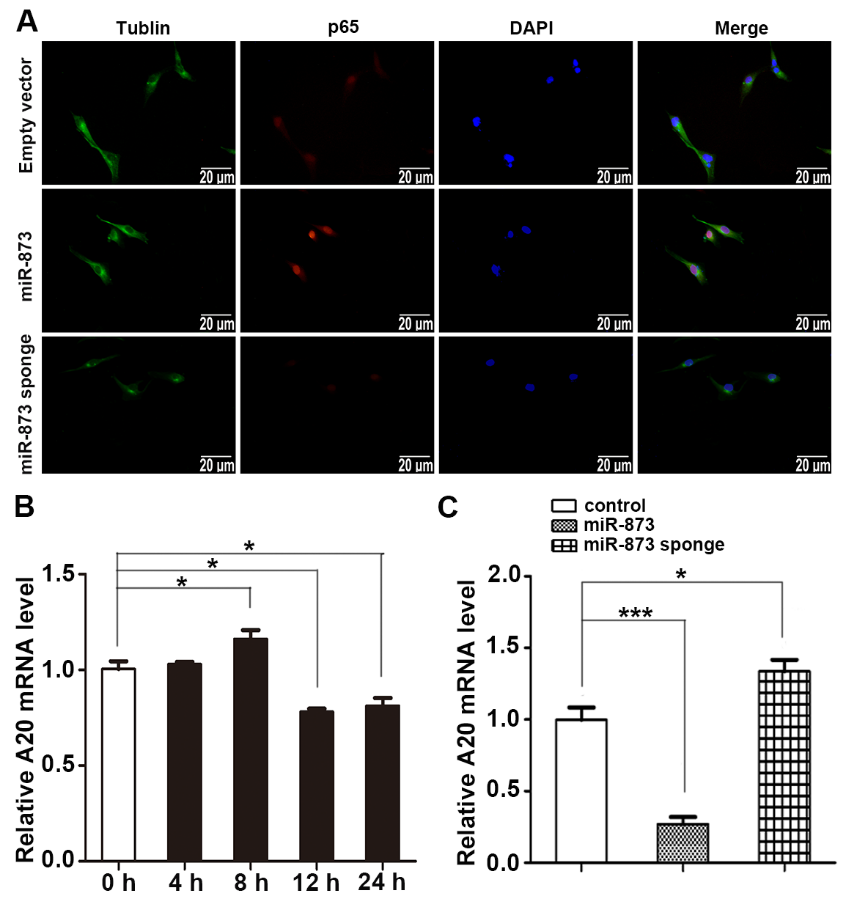


Figure S6 The translocation of the p65 protein in the nuclear was facilitated following miR-873 transfection in U87 cells. The levels of the subunit of NF-κB, p65 protein (red), were increased in human astrocytes, in which the cytoskeleton was labeled by tubulin (green) and the nuclear was marked by DAPI (blue) (A). The mRNA level of A20 was decreased following miR-873 transfection from 12 h till 24 h (B). The mRNA level of A20 was regulated following the transfection of miR-873 or its inhibitor (C). The data are mean ± S.E.M.; *n* = 3, * *p* < 0.05, *** *p* < 0.001 compared with the controls.

Table S1 The primer sequences used for RT-qPCR

| Name | primer sequence (5’-3’) | Tm (℃) | Cycles |
| --- | --- | --- | --- |
| h-ABCA1 | F: TGGAATCCTCCCTCAGTTCCT  R: GCGTGTCACTCTCATGGTCG | 60  60 | 40  40 |
| h-GCase | F: ATGGAGCGGTGAATGGGAAG  R: GTGCTCAGCATAGGCATCCAG | 60  60 | 40  40 |
| h-NPC1  h-NPC2 | F: GAGGGATTGTGGTGTTGGCT  R: TGTATCGCTCTTCAGTGGCA  F: CCCTTTCCCATTCCTGAGCC  R: GCCACTCCACCACCAGTTTT | 60  60  60  60 | 40  40  40  40 |
| h-CTSD | F: GCTGGGAGGCAAAGGCTACAA  R: TCCTGCTCTGGGACTCTCCT | 60  60 | 40  40 |
| h-A20 | F: GAAGCTTGTGGCGCTGAAAA  R: TTCCTTGAGCGTGCTGAACA | 60  60 | 40  40 |
| h-GAPDH | F: CATCACCATCTTCCAGGAGCGAGA  R: TGCAGGAGGCATTGCTGATGATCT | 60  60 | 40  40 |
| r-ABCA1 | F: GCAGCGACCATGAAAGTGAC  R: GAGGCGGTCATCAATCTCGT | 60  60 | 40  40 |
| r-A20 | F: ACGGATTCTGTGAGCGTTGT  R: GGCTCTGCCGTAGTCCTTTT | 60  60 | 40  40 |
| r-GAPDH | F: TGACTCTACCCACGGCAAGTTCAA  R: ACGACATACTCAGCACCAGCATCA | 60  60 | 40  40 |
| U6 | F: CGCTTCGGCAGCACATATAC  R: TTCACGAATTTGCGTGTCAT | 60  60 | 40  40 |
| miR-873 | F: GCGGCGCAGGAACTTGTGAG  R: CAGTGCGTGTCGTGGAGT | 60  60 | 40  40 |
| pre-miR-873 | F: GTGTGCATTTGCAGGAACTTG  R: GAACTCATCAGTCTCCTGTTCA | 60  60 | 40  40 |

**References:**

1. Koprich, J.B., et al., *Neuroinflammation mediated by IL-1beta increases susceptibility of dopamine neurons to degeneration in an animal model of Parkinson's disease.* J Neuroinflammation, 2008. **5**: p. 8.

2. Tanaka, S., et al., *Activation of microglia induces symptoms of Parkinson's disease in wild-type, but not in IL-1 knockout mice.* J Neuroinflammation, 2013. **10**: p. 143.

3. Mogi, M., et al., *Interleukin-1 beta, interleukin-6, epidermal growth factor and transforming growth factor-alpha are elevated in the brain from parkinsonian patients.* Neurosci Lett, 1994. **180**(2): p. 147-50.

4. Mogi, M., et al., *Interleukin (IL)-1 beta, IL-2, IL-4, IL-6 and transforming growth factor-alpha levels are elevated in ventricular cerebrospinal fluid in juvenile parkinsonism and Parkinson's disease.* Neurosci Lett, 1996. **211**(1): p. 13-6.

5. Nagatsu, T., et al., *Changes in cytokines and neurotrophins in Parkinson's disease.* J Neural Transm Suppl, 2000(60): p. 277-90.

6. Li, J., et al., *Tumor necrosis factor alpha mediates lipopolysaccharide-induced microglial toxicity to developing oligodendrocytes when astrocytes are present.* J Neurosci, 2008. **28**(20): p. 5321-30.

7. Bruck, D., et al., *Glia and alpha-synuclein in neurodegeneration: A complex interaction.* Neurobiol Dis, 2016. **85**: p. 262-274.

8. Shembade, N., A. Ma, and E.W. Harhaj, *Inhibition of NF-kappaB signaling by A20 through disruption of ubiquitin enzyme complexes.* Science, 2010. **327**(5969): p. 1135-9.
